# Magnetic crystals and helical liquids in alkaline-earth fermionic gases

**DOI:** 10.1038/ncomms9134

**Published:** 2015-09-09

**Authors:** Simone Barbarino, Luca Taddia, Davide Rossini, Leonardo Mazza, Rosario Fazio

**Affiliations:** 1 NEST, Scuola Normale Superiore & Istituto Nanoscienze-CNR, I-56126 Pisa, Italy; 2 Scuola Normale Superiore, I-56126 Pisa, Italy; 3 CNR—Istituto Nazionale di Ottica, UOS di Firenze LENS, I-50019 Sesto Fiorentino, Italy

## Abstract

The joint action of a magnetic field and of interactions is crucial for the appearance of exotic quantum phenomena, such as the quantum Hall effect. Owing to their rich nuclear structure, equivalent to an additional synthetic dimension, one-dimensional alkaline-earth(-like) fermionic gases with synthetic gauge potential and atomic contact repulsion may display similar related properties. Here we show the existence and the features of a hierarchy of fractional insulating and conducting states by means of analytical and numerical methods. We demonstrate that the gapped states are characterized by density and magnetic order emerging solely for gases with effective nuclear spin 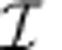 larger than 1/2, whereas the gapless phases can support helical modes. We finally argue that these states are related to an unconventional fractional quantum Hall effect in the thin-torus limit and that their properties can be studied in state-of-the-art laboratories.

## Abstract (web summary)

One-dimensional alkaline-earth-like fermionic gases with large nuclear spins and atomic repulsion host a hierarchy of states with intriguing properties. Here, the authors use analytical and numerical techniques to predict the emergence of magnetic ordering in gapped states and helical modes in gapless phases.

## Graphical abstract

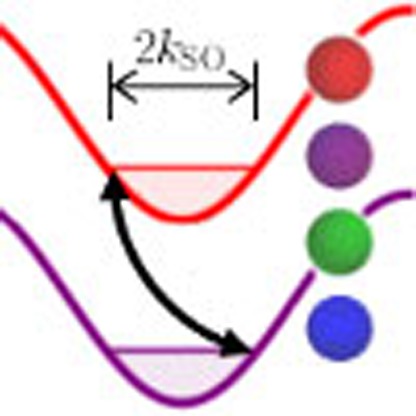

The simultaneous presence of particle–particle interactions and of (non-)Abelian gauge potentials, such as magnetic fields or spin–orbit coupling (SOC), is responsible for several spectacular phenomena, the fractional quantum Hall effect (QHE) being only the most known example. Since several years this interplay is under close scrutiny both because of its perspective role in the realization of robust quantum information protocols and because of the general interest in topological states of matter^1^. In the presence of SOC^2^,^3^, interactions can further drive the system into fractional quantum spin Hall states^4^,^5^,^6^ where edge currents are spin polarized, or other exotic phases, characterized, for example, by unusual spin textures^7^,^8^,^9^,^10^ or fractional conducting modes^11^.

Up to now, the attention has almost entirely focused on spin-1/2 (electronic) liquids, as most appropriate for the description of condensed matter systems. However, the recent progresses in the manipulation and control of cold atomic gases have brought to high relevance the study of systems of interacting fermions with a large (and tunable) spin^12^,^13^. The investigation of alkaline-earth(-like) atoms such as Ytterbium^14^,^15^,^16^,^17^ or Strontium^18^,^19^, which are characterized by a nuclear spin *I* larger than 1/2, is opening the path to the exploration of phenomena linked to the properties of SU(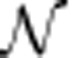) models^20^,^21^ which are not accessible with solid-state systems. The phase diagram of related multi-component Heisenberg- or Hubbard-like models has been investigated in several theoretical works^22^,^23^,^24^,^25^; yet, very little is known (see, however, ref. 26) concerning the effect of a gauge potential on an interacting system of particles with a large spin.

Synthetic gauge potentials in cold atomic systems can be induced in several ways, for example, via properly tailored laser pulses^27^ or engineered lattice shaking^28^,^29^. The implementation of these schemes has already led to the realization of light-induced magnetic fields^30^, to Rashba SOC^31^,^32^,^33^, as well as to lattice models with peculiar band structures^34^,^35^,^36^,^37^,^38^,^39^,^40^ and ladders with synthetic gauge potentials^41^. Similar approaches are suitable for application also to multi-component gases^42^,^43^, as shown in two recent spectacular experiments^44^,^45^. This fecund experimental activity, together with the rich scenario already explored for spin-1/2 systems, motivates the investigation of interacting systems with large spin coupled to gauge potentials.

Do large-*I* alkaline-earth(-like) atoms lead to mere extensions of what is already known for electronic liquids? The answer is no. These setups allow for the exploration of novel regimes that can naturally be achieved only for *I*>1/2 or through the versatility of this new playground. This puts cold-atom experiments in an excellent position for the investigation of intriguing many-body effects unattainable in conventional condensed matter setups.

In this article we consider a one-dimensional fermionic gas with an effective nuclear spin 
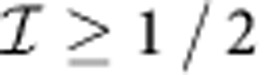
 and investigate the joint effect of interactions and of a synthetic gauge potential, which is equivalent to a Rashba SOC and an external magnetic field. Provided that the states with highest and lowest spin are directly coupled^42^,^43^, a full hierarchy of magnetic crystalline states appears at fractional fillings:


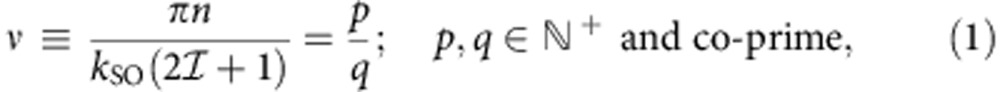


where *n* is the atomic density and *k*_SO_ is the typical momentum of the SOC (to be defined in the following). Combining analytical and numerical methods, we are able to show that these insulating phases exhibit non-trivial density and spin ordering. Furthermore, for certain fractions in equation (1) the insulating states are connected to the gapped states of an unconventional fractional QHE in the thin-torus limit (TTL)^46^. The stabilization of these gapped phases with *q*>1 requires some form of atom–atom interaction. Whereas some of them can be realized in the presence of a simple contact repulsion, it is in general true that for *p*=1, the higher the *q*, the lower the density, and thus the longer the range of the necessary interaction. These phases change dramatically once the mentioned coupling between the extremal spin states is switched off. Similarly to the spin-1/2 case^11^, the appearance of a fractional helical liquid, namely a gapless phase with low-energy excitations with a definite relation between spin and momentum direction, is reported. Our analysis, which includes also the effect of a trapping potential and of temperature, confirms that the findings of this work can be observed with state-of-the-art experimental techniques. Following the reasoning put forward in refs 42, 43, we can conclude that this setup might serve, especially in the limit of large *I*, as a quantum simulator of two-dimensional exotic interacting phases of matter in the strongly anisotropic limit.

## Results

### Model

**Figure 1:**
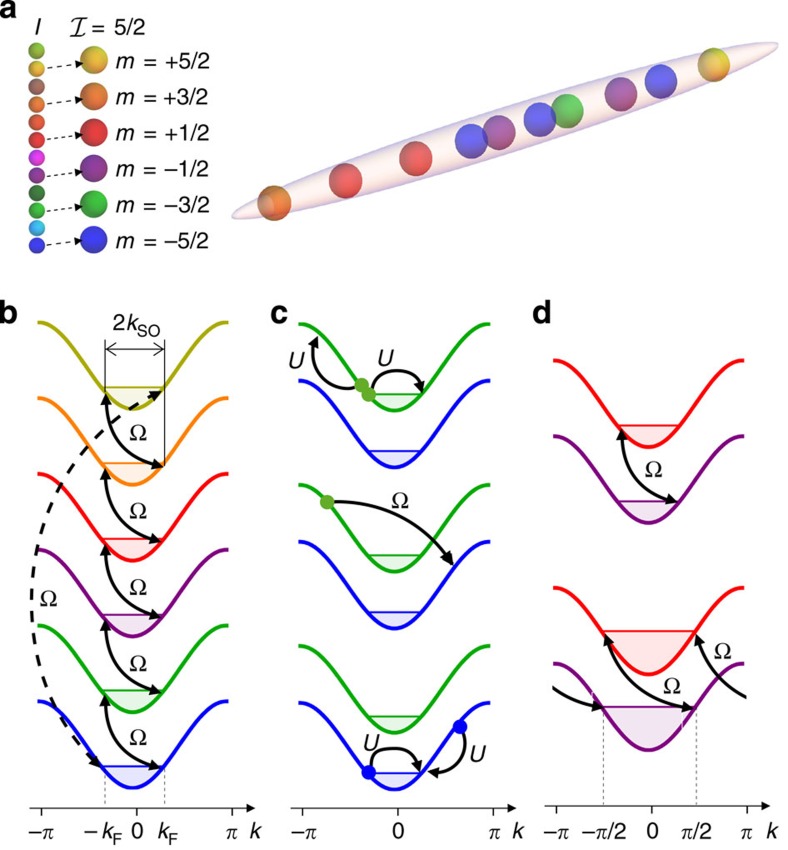
Alkaline-earth(-like) atomic Fermi gas with effective nuclear spin 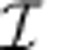. (**a**) Sketch of a one-dimensional gas of fermionic atoms with an effective nuclear spin 
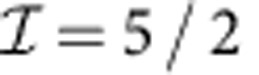
 selected from the larger set of 2*I*+1 nuclear spin states. (**b**) Energy bands of the Hamiltonian 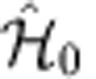 for 
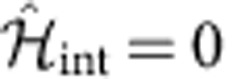
 and 
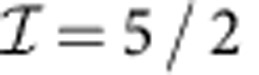
; an energy shift is inserted for representation clarity, but they are degenerate. In this case it is possible to define a Fermi momentum *k*_F_ for each spin state, so that the system has in total 
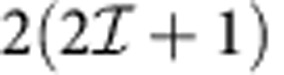
 edges. When Ω is turned on, fermions with momentum difference Δ*k*=±2*k*_SO_ and spin difference 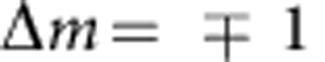 (solid arrows) or 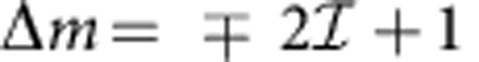 (dashed arrow) get coupled through 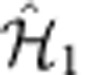 and 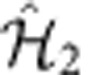, respectively: if the condition *k*_SO_=*k*_F_ is met, the system develops a full gap, corresponding to *ν*=1. (**c**) When 
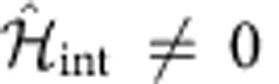
, the system can develop a gap for lower fillings *ν*=1/*q* via higher-order scattering terms. As an example, the picture highlights three intermediate processes that generate a coupling between two Fermi edges with Δ*m*=1 of a 
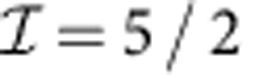
 gas: their sequence (top to bottom) originates a third-order process, which couples two Fermi surfaces for *q*=3 and *k*_F_=*k*_SO_/3. The same processes take place for any couple of edges with Δ*m*=1. (**d**) For 
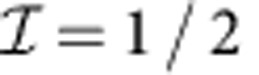
 the condition *k*_SO_=*k*_F_ is not enough because 
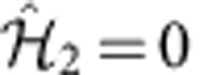
 (upper panel): when *k*_SO_=*π*/2 the identification of momenta modulo 2*π* allows for the creation of a gap (lower panel).

We consider a one-dimensional model that describes an optical lattice loaded with a gas of fermionic alkaline-earth(-like) atoms whose ground state is characterized by a large nuclear spin *I*; for a sketch, see Fig. 1a. For generality, we consider a subset of the atomic nuclear states and define an ‘effective spin' 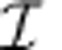, with 
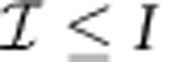
; this means that we choose a set of 
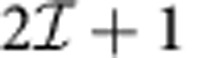
 nuclear states of the atom, labelled from now on by *m*, from the larger set of 2*I*+1 available states^16^. The Hamiltonian reads^20^


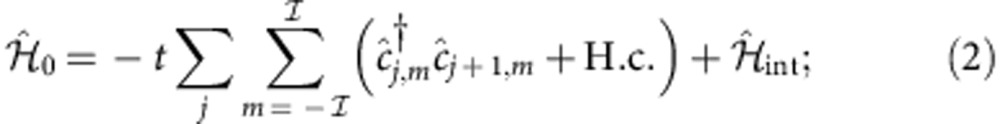


where 
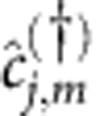
 are fermionic operators annihilating (creating) an atom at site *j* with nuclear spin *m*, and *t* is the hopping amplitude. Note that there is no inconsistency between the fermionic statistics and the study of integer 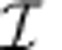, since the considered states can be selected at will from a larger half-integer manifold and, for example, need not to have adjacent nuclear quantum number^16^,^20^. 
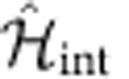
 describes an SU(
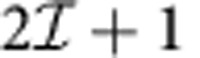
) invariant interaction, which is usually of contact kind, that is, 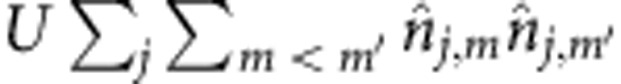, with 
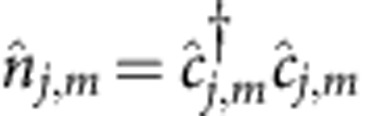
. As new proposals make the engineering of longer-range interactions in cold gases more practicable^47^, we also discuss as an example the effect of a nearest-neighbour potential, that is, 
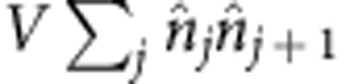
, with 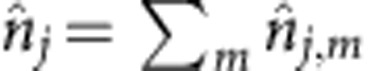.

A Raman coupling endowed with a running phase connects states that differ for Δ*m*=±1:


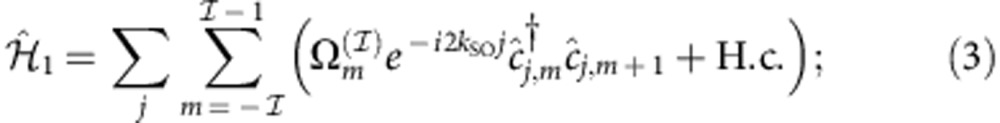


here 
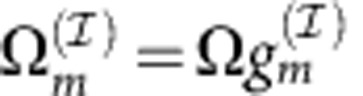
, where Ω is the Raman-coupling strength and 
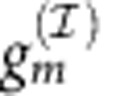
 depends on the setup. Note that the Hamiltonian 
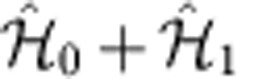
 has been experimentally implemented^44^.

The unitary transformation 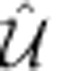 defined by 
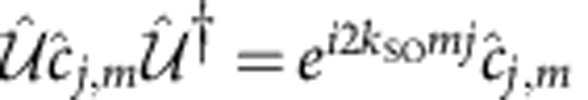
 maps the Hamiltonian 
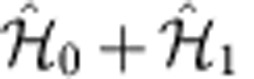
 to a spin-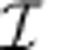 fermionic model in the presence of Rashba SOC and of a magnetic field Ω with perpendicular quantization axis (see Methods). The choice to denote the phase factor in equation (3) with *k*_SO_ becomes then clear on inspection of the kinetic term: 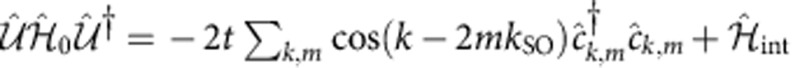.

We also consider an additional coupling between the extremal nuclear spins 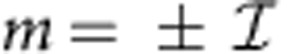, described by a Hamiltonian term 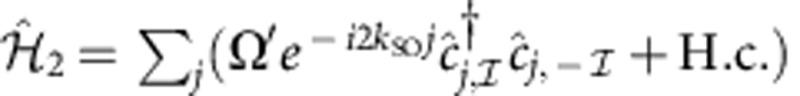. Engineering 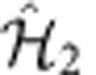 is experimentally more demanding than 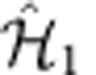 and requires a scheme where, by means of spin-dependent light shifts, the various spin states can be addressed with different frequencies. In the following we exploit the fact that, especially for small 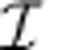, Ω′ can be tuned to the same order of magnitude of 
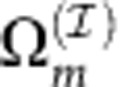
. It is important to stress that 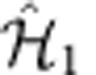 implements couplings with an open topology in spin space, namely with two effective boundaries at 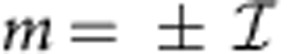. The relevant effect of 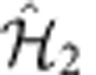 is to implement the additional coupling, which turns such topology into a closed (circular) one. The possibility to modify (and eventually switch off) 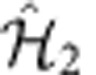 is unique to this cold-atom implementation.


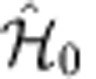
 is both invariant under SU(
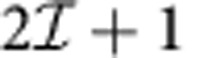
) rotations in spin space and under discrete lattice translations. For 
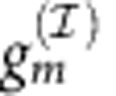
 not depending on *m*, those symmetries are broken by 
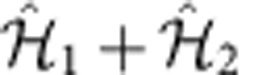
 to 
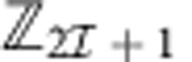
 rotations around 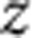 axis in spin space and to a spin-dependent discrete translation 
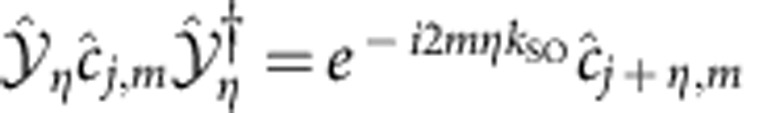
, with 
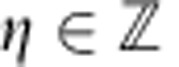
 and 
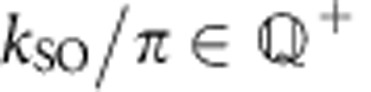
 such that 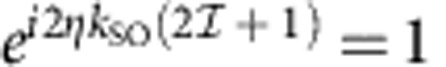.

### Analytical results on magnetic crystals for 
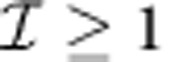


As a first step we present analytical arguments that for the fillings 
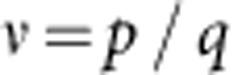
 given in equation (1) with odd *q* and for 
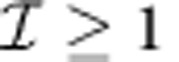
 the system described by the Hamiltonian 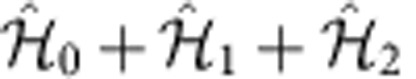 is gapped.

Gapped phases appear in the absence of interactions only for the fillings 
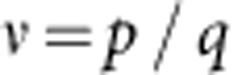
 in equation (1) with *q*=1. It is instructive to briefly discuss this case as it helps understanding the interacting one. By writing 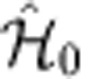 in momentum–space representation it is possible to define a Fermi energy and a Fermi momentum *k*_F_ relative to the case with Ω=0 for each spin state *m* (see Fig. 1b). On the other hand, 
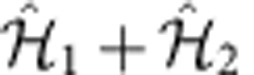
 couples modes with 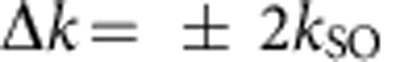 and 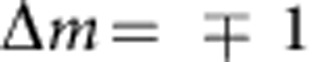 or 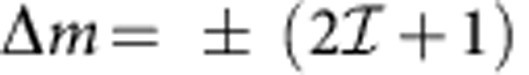: whenever *k*_SO_=*k*_F_, a fermion can be simultaneously spin flipped and scattered from one edge of the Fermi sea to the other one. It is a well-known fact that these processes lead to the opening of a gap at those edges, namely the involved modes at 
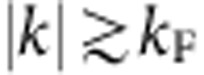
 get an energy increase of order ∼Ω with respect to those at 
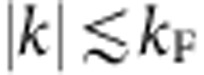
. The implementation of couplings with a periodic topology in spin space ensures that any edge of the Fermi sea gets coupled (see Fig. 1b) and develops a gap. Thus, the whole system develops a full gap. In this case, the density *n* is 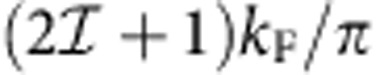, so that *ν*=1. Since higher-order processes generated by 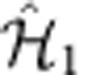 and 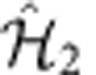 can connect spin states with |Δ*m*|>1, a full gap opens whenever *k*_F_=*pk*_SO_, *p*∈[1, 2,…, 2*I*]: these phases correspond to *ν*=*p*.

For values of *q* larger than 1, insulating phases are determined by the joint action of 
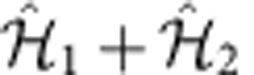
 and 
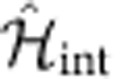
, representing the interplay of the gauge potential and interactions. The situation is a generalization of the previous case. As an example, let us consider the case *p*=1 and *q*=3 depicted in Fig. 1c; the system is in a low-density situation and *k*_SO_=3*k*_F_. Thus, 
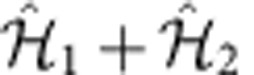
, which couples momentum states with Δ*k*=2*k*_SO_, cannot induce any direct coupling between the Fermi edges at ±*k*_F_ as before. However, there can still be an indirect coupling in the presence of interactions because the latter can scatter two particles initially at the same Fermi edge with momentum *k*_F_, to the modes with momentum states *k*_F_ (the other Fermi edge) and 3*k*_F_ (spin is unchanged). In this case, if 3*k*_F_=*k*_SO_, a new resonance condition is met and a coupling between Fermi edges develops through high-order processes mediated by interactions. The effective coupling is obtained via the transfer of three particles across the Fermi surface (two transfers are due to interactions and do not change *m*, one is induced by the Raman coupling and changes *m*; see Fig. 1c). As in the case *q*=1, the two gapless excitations at the Fermi edges involved in each of these processes become gapped excitations, with gap, which is now of order ∼*U*^2^Ω. The system develops a full gap because any edge of the Fermi sea gets coupled and gapped.

The existence of gapped phases for *q*>1 and odd can be put on a more solid ground using the bosonization technique, which is the natural analytical tool to take into account interactions in one dimension. In the next paragraph we present the key ideas that lead to the emergence of the gapped phases; details are in the Methods.

By linearizing the non-interacting spectrum of 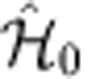 around the Fermi energy, we can write 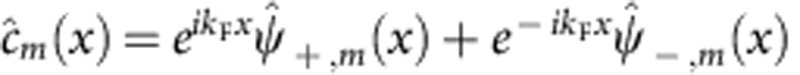, where 
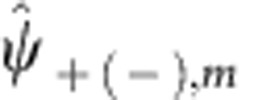
 is the right (left)-moving operator of the *m*-th nuclear spin state. 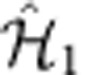 and 
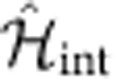
 originate 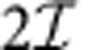 processes of the form


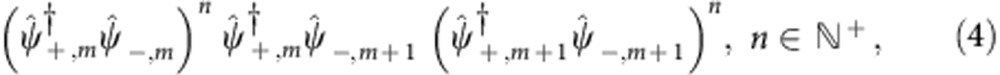


with 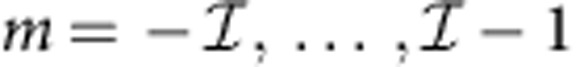, which are relevant only when momentum is conserved, that is, at *k*_F_=*k*_SO_/*q* with *q*=2*n*+1. When *n*=1, the term (4) reproduces the low-energy physics of the processes discussed before and displayed in Fig. 1c. 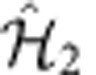 generates one more relevant process, expressed in the form of equation (4) by replacing *m* with 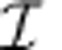 and *m*+1 with 
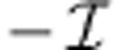
. Hamiltonian 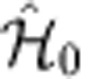 and terms in equation (4) must be treated with bosonization; the analysis, to be carried out according to refs 48, 49, shows that they can make the system fully gapped (see Methods). It is important to stress that, given a specific filling, a generic interaction does not necessarily stabilize a gapped phase. Explicit cases are discussed in the following.

### Numerical results on magnetic crystals for 
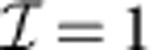


To fully characterize the properties of this hierarchy of gapped phases, we perform numerical simulations for the simplest case 
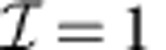
. We employ the density-matrix renormalization group (DMRG) algorithm^50^ that in one dimension provides essentially exact results (see Methods) and allows us to explore the cases of even *q* and *p*>1, which are not easily accessible with bosonization. We take for simplicity 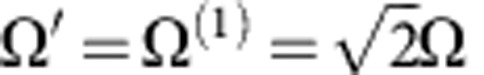 (
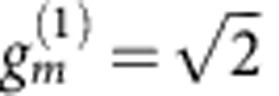
 and is independent of *m*).

**Figure 2:**
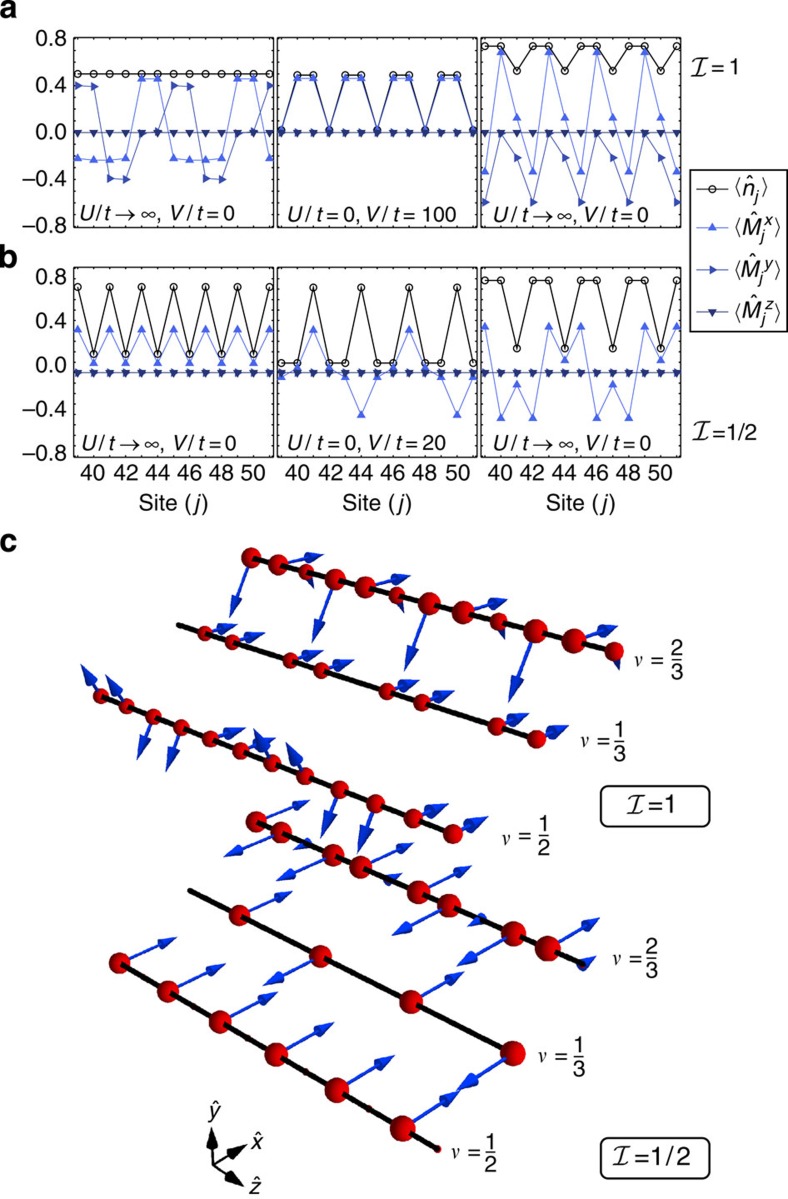
Density and magnetic order of the magnetic crystals. Density 
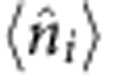
 and magnetic order 
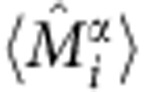
 of the fractional phases at 
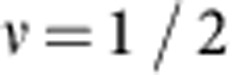
, 
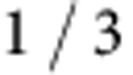
 and 
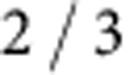
 (from left to right) for: (**a**) 
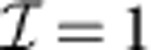
, *k*_SO_=*π*/3 and (**b**) 
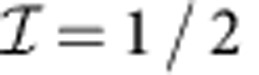
, *k*_SO_=*π*/2 as obtained from DMRG simulations of a system of length *L*=96 with 
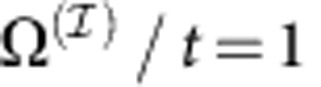
. For the interaction parameters see the panels. Since the system is a crystal with small boundary effects, for a better readability we only plot its central part. (**c**) Sketch of the density (red balls with radius related to 
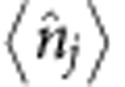
) and magnetic properties (blue arrows representing the vector 
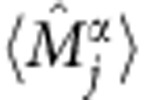
) of the insulators at 
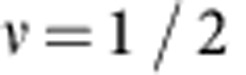
, 
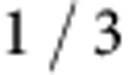
 and 
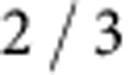
 (from left to right) for 
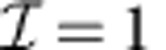
 and 
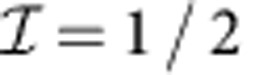
.

The results in Fig. 2a are related to the fillings 
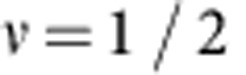
, 
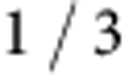
 and 
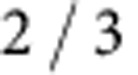
 with *k*_SO_=*π*/3 and display the density profile 
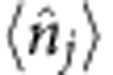
 and the magnetization 
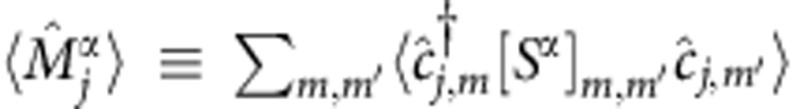
, where *S*^*α*^ is a 
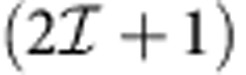
-dimensional representation of the SU(2) spin operator (*α*= *x*, *y*, *z*). The plots show that the incompressible phases are characterized by both density and magnetic order.

Let us begin with the density order, considering, for example, the case 
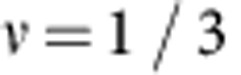
; here a nearest-neighbour interaction stabilizes a density wave with one particle every three sites. Note that in the usual SU(3) Hubbard model (Ω=0, no gauge potential) a nearest-neighbour interaction only stabilizes a density wave with at least one particle every two sites. Conversely, for *U*,*V*=0 (no interactions) no gapped phases exist at this filling. More generally, the simultaneous presence of interactions and of the (commensurate) SOC proves to be crucial in all the cases considered in Fig. 2a both for the opening of the gap and for the crafting of the properties of the resulting insulator.

Concerning magnetic order, in all the cases considered in Fig. 2a we observe that 
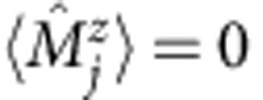
, which implies that in every site the ±*m* nuclear-spin components have the same occupation numbers. Magnetization thus lays in the *x*–*y* plane and winds as a function of position in ways that depend on the filling (see the sketches in the upper panel of Fig. 2c for 
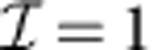
).

**Figure 3:**
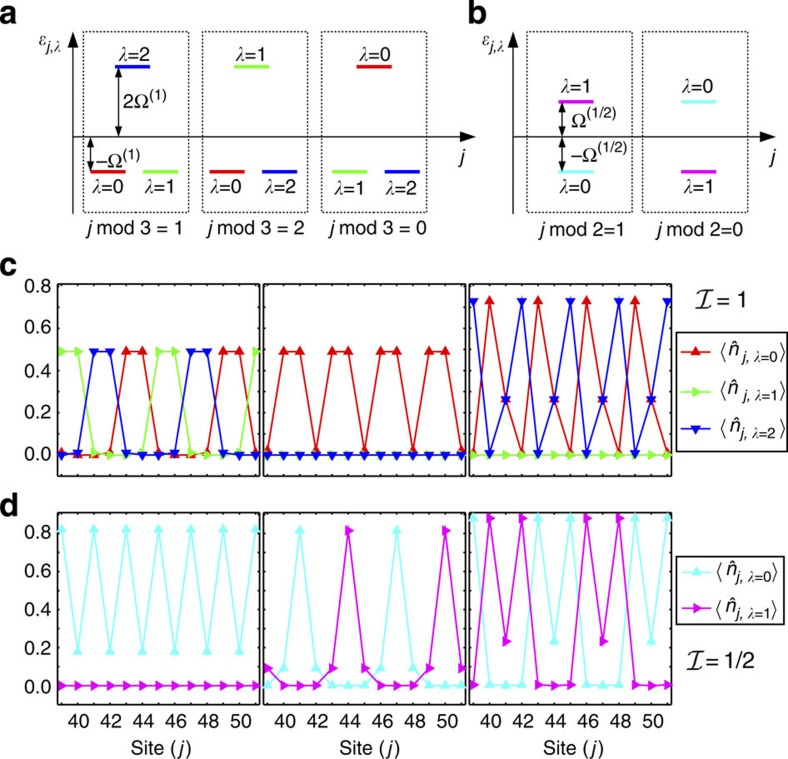
Magnetic crystals. Energy-spin structure *ɛ*_*j*,*λ*_ for: (**a**) 
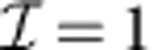
 and *k*_SO_=*π*/3, (**b**) 
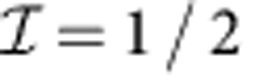
 and *k*_SO_=*π*/2. Density plots 
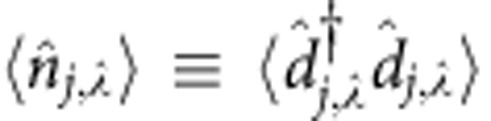
 at 
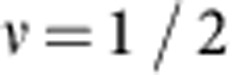
, 
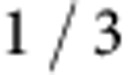
 and 
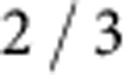
 (from left to right) for: (**c**) 
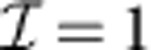
 and (**d**) 
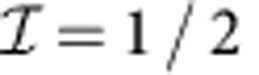
 obtained through DMRG simulations (see the caption of Fig. 2 for the parameters).

The density and magnetic ordering found in the DMRG simulations becomes transparent by considering the limit Ω^(1)^/*t*≫1, Ω^(1)^/*U*≫1. Since the discussion of this limit easily generalizes to every value of 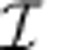, provided only that 
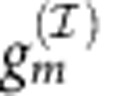
 is independent of *m*, we discuss it in full generality, although for the present case 
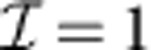
. The Hamiltonian 
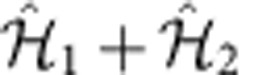
 is invariant under the 
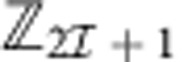
 group related to the on-site transformation 
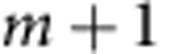
 (here 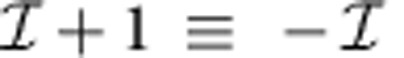), and is diagonalized by 
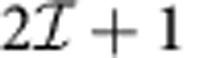
 local eigenmodes of the form:


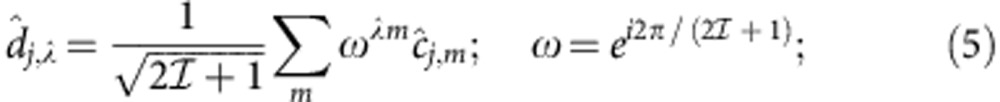


with 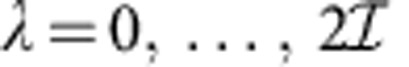. As 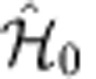 is SU(
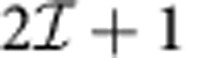
) invariant, it is unaffected by this basis change. However, the on-site energy of each transformed spin state depends both on the site *j* and on *k*_SO_: 
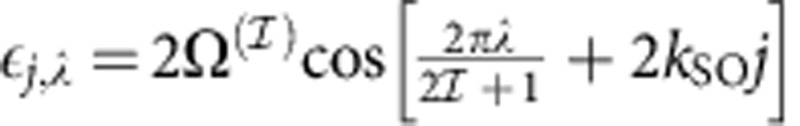
 (see the plot in Fig. 3a for 
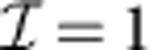
 and *k*_SO_=*π*/3).

If for every site one only considers the states with lowest energy, one intuitively realizes that when the mean interparticle space *n*^−1^ is commensurate with the space periodicity of *ɛ*_*j*,*λ*_, gapped density waves can appear. In addition, atoms will be magnetized according to the spin state 
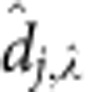
, which minimizes the energy at site *j*. We argue that this is the nature of the states that are reported in Fig. 2a and that we have accordingly dubbed ‘magnetic crystals'.

To substantiate our claim, in Fig. 3c we compute 
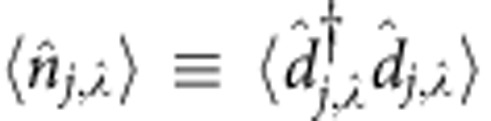
 for the same states observed in Fig. 2a (the reader can easily verify that 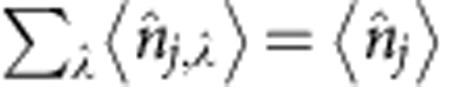). The clear patterns plotted in the three panels of Fig. 3c demonstrate that equation (5) identifies the best spin basis for understanding this problem, even if we work in the limit Ω^(1)^/*t*∼1. To further elaborate on this point, let us consider the large *U* limit for *k*_SO_=*π*/3 with an average density of one particle every two sites (
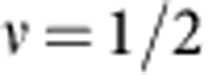
). In the absence of hopping the ground state is highly degenerate. With a small, finite, hopping the system will lower its energy by delocalizing the particles. This tendency is however strongly suppressed by the large on-site energy *U*. The resulting competition leads to the formation of dimers locked together to form a crystal as shown in Fig. 3c (for other values of *k*_SO_ see the Supplementary Figs 1 and 2 and the Supplementary Note 1).

This simple model provides a physical intuition of the fact that 
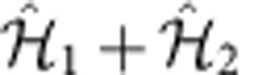
 induces magnetic crystals at the fillings in equation (1), thus complementing the information provided by bosonization for odd *q* and by DMRG for the other considered cases. The different magnetic properties in the *x*–*y* plane of the states with different *λ* determine the patterns observed in Fig. 2. Moreover, the model shows that the crystals break the symmetry 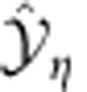, since 
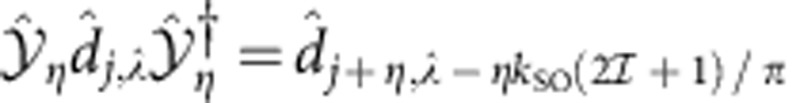
 and are thus degenerate with other equivalent configurations obtained through the action of 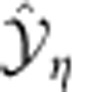.

In the Methods we quantify the gap protecting these states, which supplies a temperature range where the defining properties of these systems can be observed. In addition, a gap protects the existence of the magnetic crystal in the presence of a slight breaking of the 
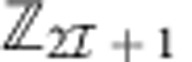
 symmetry due to the 
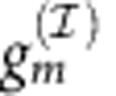
. We also present numerical simulations in the presence of a trap, showing that for properly tuned densities, the typical patterns of the mentioned insulators can still appear. In addition, we elaborate on the possibility of measuring the defining features of such insulators.

### Magnetic crystals for 
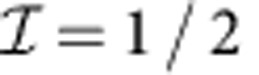


The case 
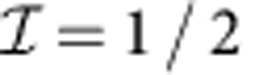
 is peculiar and needs to be considered separately. Here 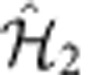 induces an additional coupling between states that are already coupled by 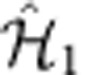. For this case we thus set 
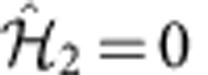
 (see ref. 51 for the study of the model at high filling, *n*=1). Let us begin by considering the non-interacting case and the filling *ν*=1. In the presence of 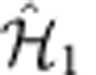, only two of the four gapless modes of the non-interacting system are coupled and become gapped^52^. Exploiting the presence of a lattice, it is however possible to invoke the equivalence of momenta on shifts of 2*π* and hence to derive the additional condition *k*_F_+2*k*_SO_=−*k*_F_+2*π* to force the two remaining gapless modes to become gapped. Together with *k*_SO_=*k*_F_ it ensures that the system enters a gapped phase at *ν*=1 (see Fig. 1d). Contrary to the previous case, *k*_SO_=*π*/2 and thus the resulting gapped phase is a lattice effect, without a proper continuum limit.

Bosonization can provide an analytical description of the mechanisms that lead to the appearance of the gapped phases for interacting systems and shows that, similarly to the non-interacting case, *k*_SO_=*π*/2 is a necessary condition for *p*=1 and *q* odd. Avoiding unessential details, we only mention that through the explicit study of the 
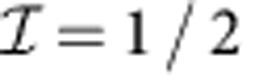
 case, for which precise mappings between the microscopic interacting model and the bosonization parameters are known, in the Methods we argue that the higher the value of *q*, the longer the range of the interactions required to let the gapped phase appear (see Supplementary Figs 5 and 6 and Supplementary Note 2 for additional information on the case *k*_SO_≠*π*/2 and on the gapping mechanism).

DMRG simulations for 
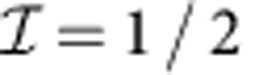
 are shown in Fig. 2b; in this case the Hamiltonian is real and thus 
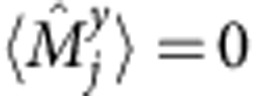
: the magnetization develops along the 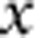 axis only (see the sketches in Fig. 2c for 
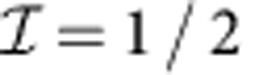
). Again, this behaviour can be understood through the analysis of the model for Ω/*t*≫1 and Ω/*U*≫1 (
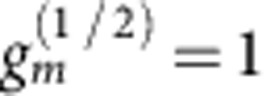
): for 
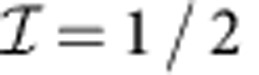
, the modes 
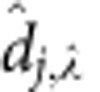
 are the eigenstates of 
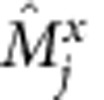
 (see Fig. 3b,d).

### Helical liquids

According to the previous discussion, for 
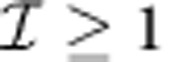
 gapped phases at the fillings in equation (1) arise in the interacting system only when 
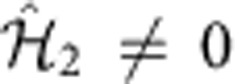
 and, for 
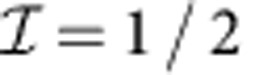
, when *k*_SO_=*π*/2 (lattice effect). More generally, explicit inspection has shown that even when 
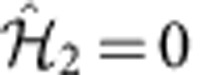
 the condition 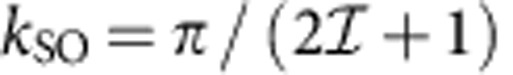 for 
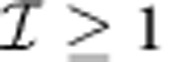
 can open a full gap through a high-order process, which exploits the presence of a lattice (that is, of momenta identification on shifts of 2*π*). When these conditions are not met, and thus the full gap does not develop, we show that for the fillings in equation (1) the system described by 
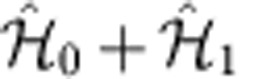
 is a helical liquid^11^.

An intuition for this phenomenology is again provided by the non-interacting case 
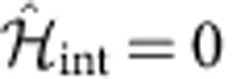
 and *q*=1. Here the two Fermi edges at 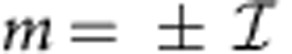 and *k*=±*k*_F_, respectively, remain unperturbed (see Fig. 1b) and represent two gapless helical fermionic modes, which are the lowest-energy excitations of the system. Bosonization techniques applied to the case 
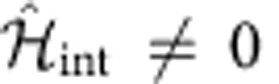
 for *q*≥1 and odd clearly pinpoint the helical nature of the low-energy spectrum, although the gapless modes for *q*>1 are linear combinations of the original modes. In addition, their conductance is fractional^11^: these two properties define a fractional helical liquid. Similarly to the magnetic crystals, bosonization reveals the existence of requirements on the range and intensity of the atom–atom repulsion.

Numerically we cannot fully access the helical nature of the first excitations; we rather diagnose the existence of a helical ground state through two observable quantities. First, as a consequence of the existence of two gapless modes, the low-energy spectrum is described by a conformal field theory in 1+1 dimensions with central charge *c*=1. Second, the system is characterized by a current pattern 
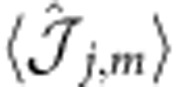
, where 
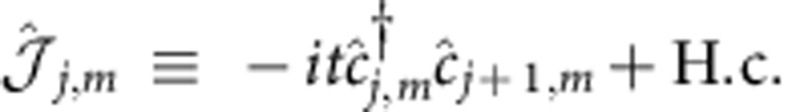
, which is different from zero and features a flow direction related to the sign of *m*.

**Figure 4:**
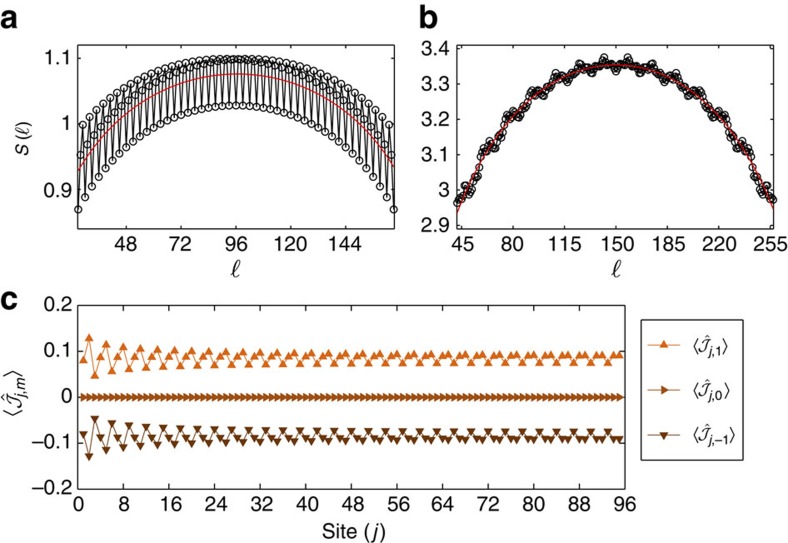
Helical liquids. DMRG simulations of the entanglement entropy of the state for 
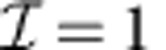
, *k*_SO_=*π*/3, 
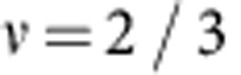
, Ω^(1)^/*t*=1 for: (**a**) *U*/*t*→∞, *L*=192 and (**b**) *U*/*t*=0, *L*=300. Thin red lines are fits with the Calabrese–Cardy formula, which yield: (**a**) *c*=1.0±0.2 and (**b**) *c*=2.94±0.06. (**c**) Helical currents 
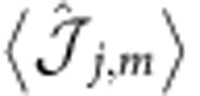
 for the case Ω^(1)^/*t*=1. For clarity, only the first half of the chain is plotted.

Our numerical results for the case 
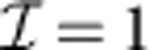
, *k*_SO_=*π*/3 and 
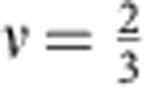
 are shown in Fig. 4. For ground states of theories with a low-energy conformal limit, the Calabrese–Cardy formula^53^ predicts that the entanglement entropy 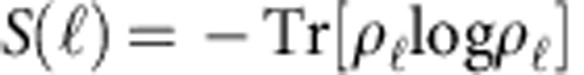, obtained through a bipartition of the system into the two blocks 
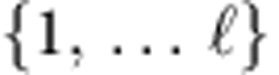
 and 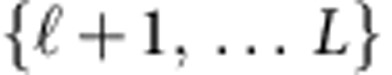 (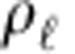 is the reduced density matrix of the block of size 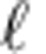), should be proportional to *c*: 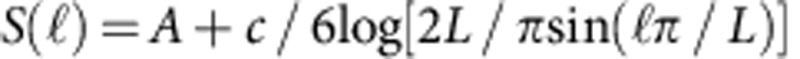 (for open boundary conditions). Figure 4a shows 
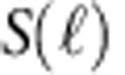
 as computed through DMRG simulations for *L*=192 and a fit with the previous formula leaving *A* and *c* as free parameters. The result clearly indicates *c*=1 (see Supplementary Fig. 4 and Supplementary Note 1 for another case). The current profile 
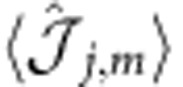
 displayed in Fig. 4c shows a finite saturated value for *j*∼*L*/2 and *m*=±1 (note that they flow in different directions); for *m*=0 the absence of a current is imposed by symmetry reasons. The oscillations are a boundary and finite-size effect and decay to zero for *L*→∞ (see Supplementary Fig. 3 and Supplementary Note 1). Note that in this case, even if 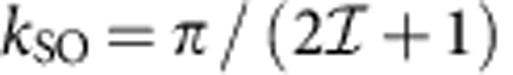, the system is gapless and thus amenable to the study of helical properties.

The simultaneous presence of two gapless modes (*c*=1) and of helical current patterns, indicating the presence of a helical state, results from the interplay between interactions and the gauge potential. In the non-interacting case, at this filling the system has three gapless edge modes, which reflect in a central charge *c*=3, as shown in Fig. 4b. On the other hand, if we tune Ω=0 (and thus *k*_SO_ disappears from the Hamiltonian), the model is equivalent to a SU(3) Hubbard model, which cannot develop helical currents, since the Hamiltonian is real. Even the milder request Ω≠0, *k*_SO_=0 results in a state without helical currents for the same reason.

## Discussion

In this article we have provided an analytical and numerical study of spin–orbit coupled alkaline-earth(-like) gases and demonstrated the existence of a full hierarchy of gapped and gapless phases with exotic properties. We have characterized several different behaviours ranging from magnetic crystals to helical phases^11^, identifying the effect of the simultaneous presence of interactions and gauge potentials. The discussed phases can be experimentally realised in state-of-the-art cold-atom laboratories.

An interesting and alternative interpretation of our results in terms of an unconventional fractional quantum Hall effect in the TTL follows from noticing that 
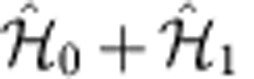
 can be interpreted as a spinless fermionic model with one (finite) synthetic dimension coupled to a synthetic magnetic field^42^,^43^. The presence or absence of 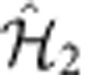 corresponds to different boundary conditions along the synthetic dimension, a feature that is relevant only for 
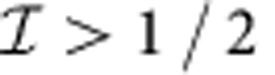
 because, for 
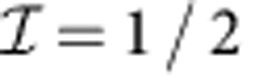
, Hamiltonian 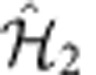 connects states that are already linked by 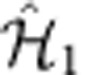. Thus, one-dimensional alkaline-earth(-like) gases provide access to the physics of (quasi-)two-dimensional models through the mapping of the nuclear spin states to a (finite) synthetic dimension. In the system considered in this article, the synthetic lattice is pierced by a magnetic gauge potential with flux per plaquette Φ/Φ_0_=*k*_SO_/*π* (Φ_0_ is the quantum of flux)^43^.

For 
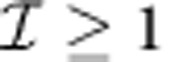
 and 
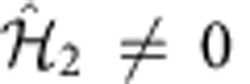
, the effective geometry of the system is that of a narrow cylinder, since the synthetic-dimension length, 
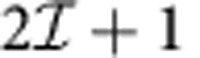
, is much smaller than the real-space one. In this limit, usually called the TTL^46^,^54^, the fractional quantum Hall states are density waves, with features coinciding with those presented in this article. First, the filling *ν* coincides with the ratio *N*/*N*_Φ_ between the number of particles and the number of magnetic fluxes piercing the synthetic lattice, which is the well-known condition for observing the Laughlin series in QHE. Second, the density waves of the TTL for *ν*=1/*q* display one particle every *q* sites, similarly to what we find in Fig. 2. In addition, the helical properties that appear for 
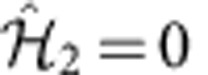
, that is, in an effective stripe geometry, can be interpreted as precursors of the edge modes of the QHE^43^. This fecund analogy is lost for 
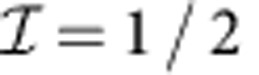
 (or for spin-1/2 quantum liquids, such as degenerate electron gases): in a two-leg geometry, there is no difference between a cylinder and a stripe. Our study therefore suggests that alkaline-earth(-like) atoms are a promising tool for bringing ultra-cold atomic gases into the quantum Hall regime, a long-standing and yet to be achieved goal, through the access of its thin-torus/stripe limit.

The appearance of the phenomenology that we have just described is *a priori* unexpected. Indeed, the SU(
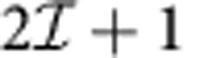
)-invariant interaction is, in the quasi-two-dimensional picture, strongly anisotropic (short-ranged along the chain and infinite-ranged in the synthetic direction), and does not resemble the features neither of the Coulomb nor of the contact repulsion, which are usually considered in the QHE theory. Spin–orbit coupled alkaline-earth(-like) gases are a natural quantum simulator of the physics of the fractional QHE in an array of quantum wires^48^,^49^. More precisely, because of the unusual properties of the interaction, we are dealing here with the TTL of an unconventional form of QHE, and it is an exciting perspective to investigate up to which point it shares features with the standard QHE. More generally, large-*I* Fermi gases provide a valuable experimental toolbox for the study of two-dimensional exotic phases of matter through coupled arrays of one-dimensional systems^48^,^49^,^55^,^56^,^57^,^58^. Moreover, even if in this work we have explicitly addressed the SU(
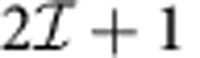
) case as relevant for alkaline-earth-(like) atoms, we argue that our predictions should extend to the situations where the SU(
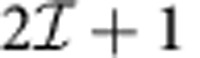
) symmetry is only slightly broken, such as in several alkaline atomic gases.

The unprecedented versatility of these setups motivates further speculations and research: we leave, for example, as an interesting open perspective the extension of this study to multi-component bosonic systems^59^,^60^,^61^,^62^,^63^,^64^,^65^,^66^.

## Methods

### Relation with Rashba SOC

Let us first explicitly display that the unitary transformation 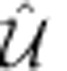 defined in the Results leads to a Hamiltonian, which is formally equivalent to a Rashba SOC model:


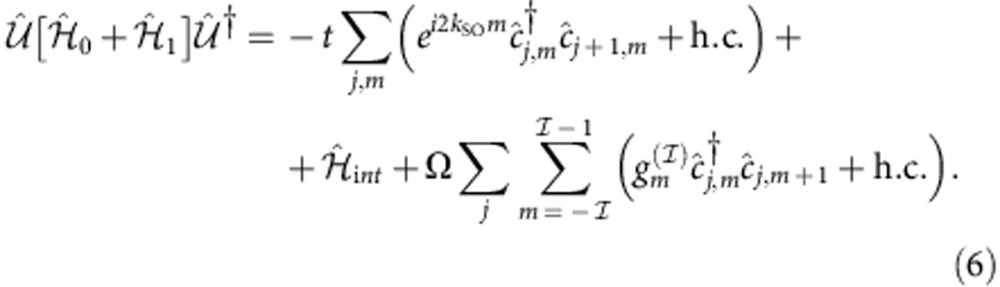


We have already commented on the fact that the term proportional to *t* is the lattice version of a Rashba SOC. The term proportional to Ω is related to a magnetic field applied along the 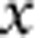 direction, perpendicular thus to the quantization axis of the SOC; if 
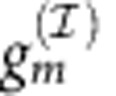
 assumes the value of the proper Clebsch–Gordan coefficient, the equivalence is formally tight. Concerning interactions, it is natural to assume that 
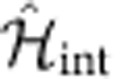
 depends only on the density operators 
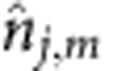
, which are left unchanged by 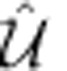; thus: 
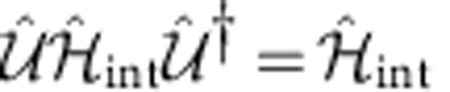
.

### Bosonization

In the following we briefly discuss the existence of the magnetic crystals within the bosonization framework. The discussion follows the guidelines set in refs 11, 48, 49. Central to the bosonization technique is the expression of 
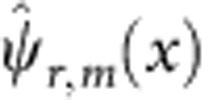
 (see the Results for the definition) as 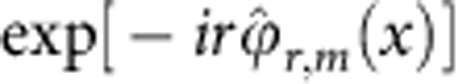, *r*=±, where the bosonic fields 
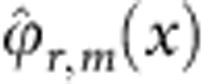
, satisfy 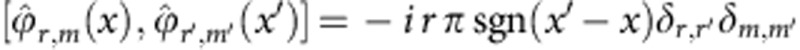. We linearize the non-interacting part of 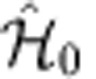 close to the Fermi energy and subsequently introduce density–density interactions, such as 
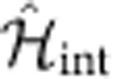
; the low-energy part of the full 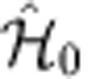 can then be cast into the quadratic form


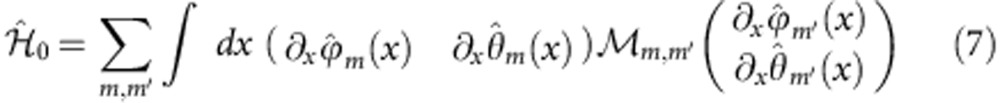


with 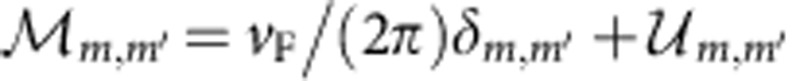; *v*_F_ is the Fermi velocity, and 
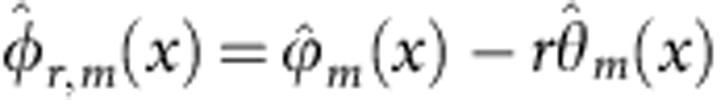
; 
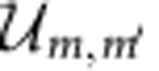
 describes the scattering processes induced by 
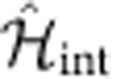
 only.

To study the interplay of 
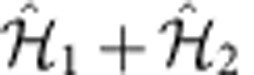
 and 
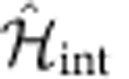
, we formally rewrite the fermionic operator as


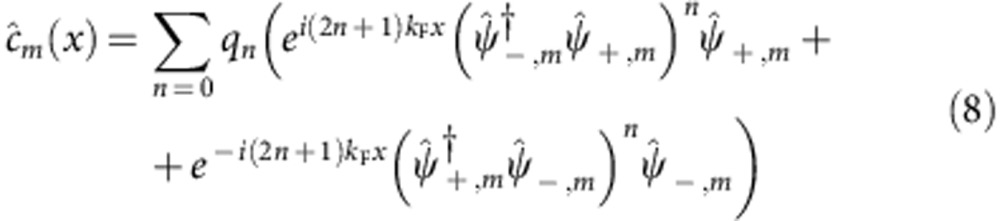


which takes into account the non-linearities of the free spectrum, particularly important when interactions are strong enough; *q*_*n*_ are unknown coefficients. Applying equation (8) to a single-term 
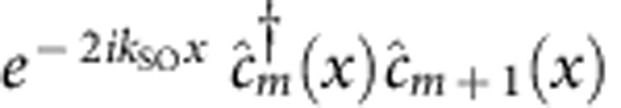
 of 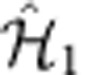 or to 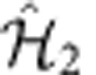 we get several contributions, of which those in the form:


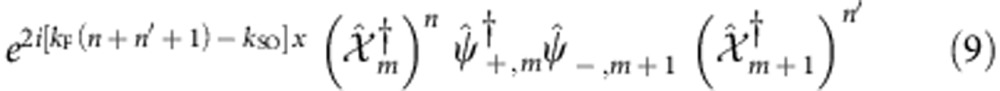


are of special interest. Here we have introduced the notation: 
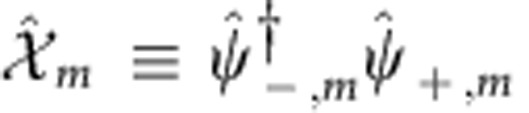
; 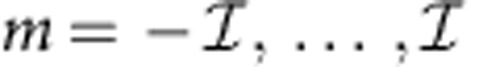 (with 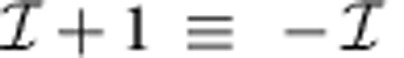). The terms (9) are the only ones that conserve momentum independently on the value of *k*_SO_, provided that


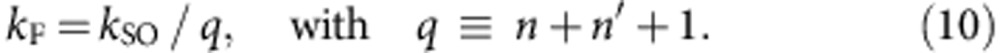


In this case, when *n*=*n*′ (and thus *q*=2*n*+1 is an odd integer), terms (9) coincide with those in (4).

The bosonized version of such operators is:


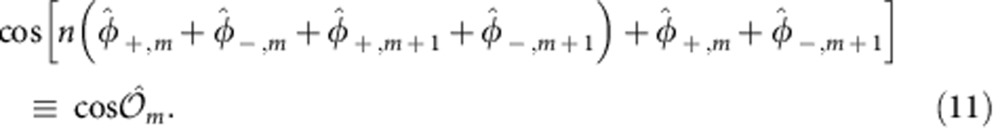


Note that 
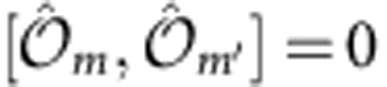
 for 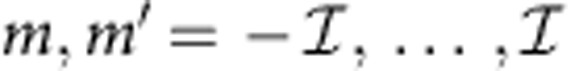. When the operators 
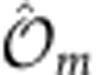
 are relevant in the renormalization group sense (here we assume 
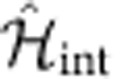
 to be such that this is true), they minimize the quantity 
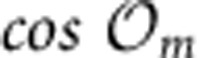
 and in a semi-classic approach 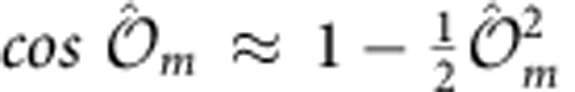. Each of these terms donate a gap to two originally gapless modes, so that the gapless Luttinger Hamiltonian (7) becomes fully gapped.

The existence of fractional phases with *q* even (*n*′=*n*±1) cannot be straightforwardly explained using bosonization. Let us consider as an example the case *q*=2. For each term (9) we can choose *n*=0 and *n*′=1 but also *n*=1 and *n*′=0, so that 
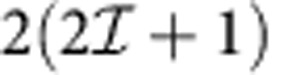
 sine-Gordon terms with non commuting arguments and with the same scaling dimension appear. In this case a semi-classic approach cannot be used and the solution of the resulting Hamiltonian is an interesting open question.

In addition, we mention that the bosonization framework can be used to show the existence of fractional insulating phases for *p*>1 and *q* odd, by introducing a fictitious coupling between different nuclear spin states of the form^48^:


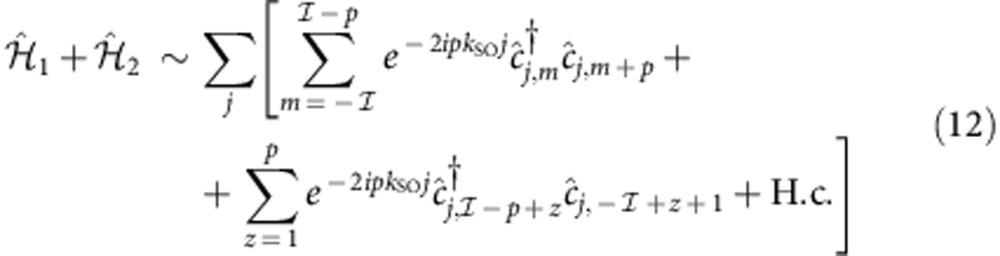


which models higher-order couplings between spin states with Δ*m*≠±1. Bosonization of this term yields results that are completely equivalent to the previous ones, apart from the fillings at which gapped phases appear.

When we consider atoms with two nuclear spin states, namely 
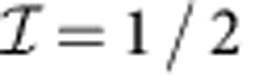
, we set 
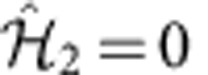
. Gapped phases at fillings *ν*=1/*q* with odd *q* are stabilized by (9) and by


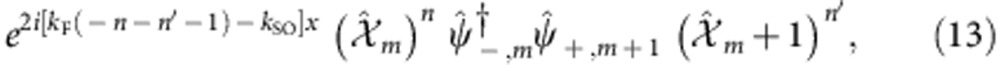


that can be derived following the same procedure used for the terms (9). With *m*=−1/2, they imply *k*_*S*O_=*π*/2 and *k*_F_=*π*/(2*q*), respectively. If we introduce the charge (*c*) and spin (*s*) bosonic fields 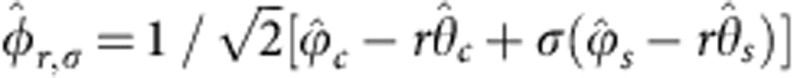 the Hamiltonian 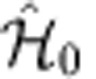 can be written as


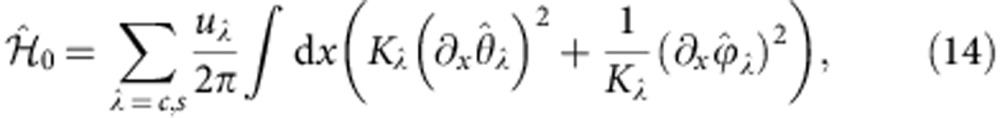


*K*_*λ*_ is the usual Luttinger parameter that takes into account the strength of the interaction, *u*_*λ*_=*v*_F_/*K*_*λ*_ is the renormalized Fermi velocity. On the other hand the terms (9) and (13) assume a bosonized form proportional to


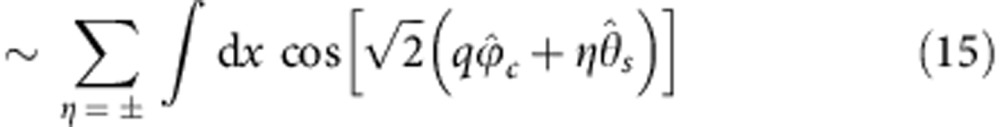


they couple the charge and the spin degrees of freedom. A renormalization-group calculation shows that they are relevant when *K*_*c*_<3/*q*^2^, assuming a SU(2) invariant interaction, that is, *K*_*s*_=1 (ref. 11). Physically, this means that an on-site repulsive interaction, for which 1/2≤*K*_*c*_<1 (ref. 67), cannot stabilize a gapped phase for *q*≥3. Longer-range interactions are thus necessary (for nearest-neighbour repulsion, for instance, it is possible to achieve *K*_*c*_<1/2 (ref. 67)), and in general we expect that, even for 
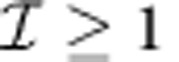
, the higher the value of *q*, the longer the range of the interactions required to open the gap.

### Experimental issues

**Figure 5:**
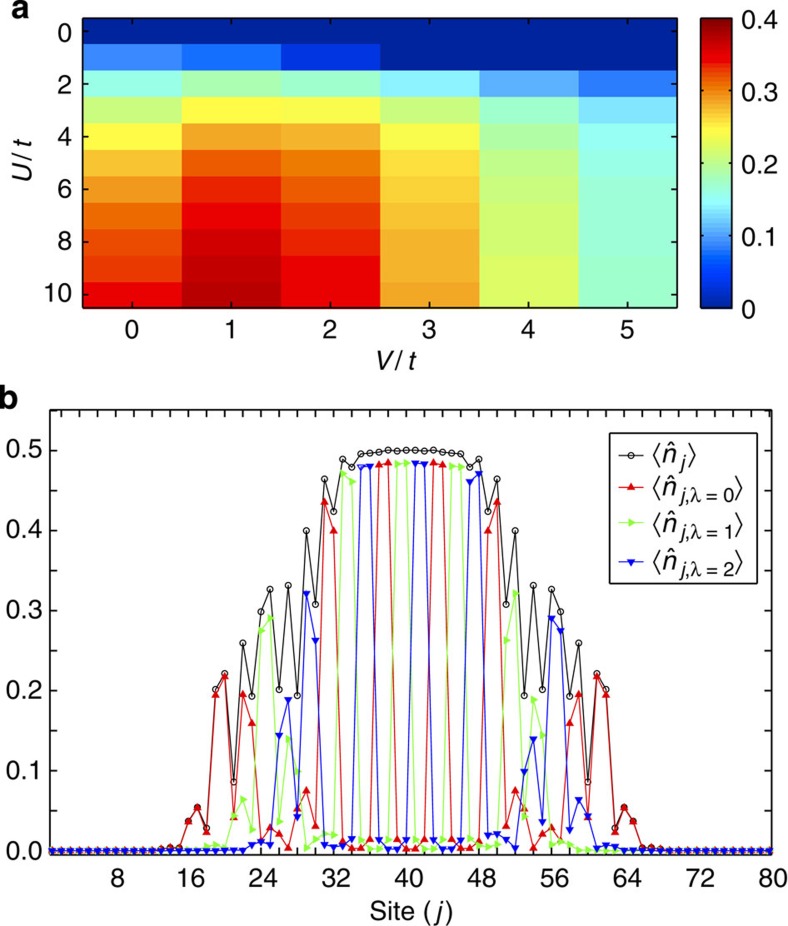
Robustness of the gapped phase. (**a**) Energy gap (units of *t*) of the insulating phase at 
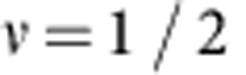
 and 
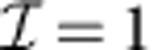
 as a function of *U*/*t* and *V*/*t* computed through exact diagonalization for a system of *L*=6. The parameters of the simulation are Ω^(1)^/*t*=1 and *k*_SO_=*π*/3. For larger values of *U*/*t* the gap saturates. (**b**) Density profile for the same system in the presence of a harmonic confinement with 
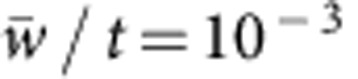
. The simulation is performed for 16 fermions, *U*/*t*=10 and *V*/*t*=0. In the centre of the system the typical density and magnetic order of the insulator with filling 
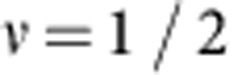
 appear.

The gapped phases in Fig. 3 at 
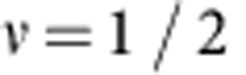
 and 
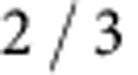
 are stabilized by the solely on-site repulsion, and thus accessible in current experimental settings^16^,^44^. It is therefore important to check for the presence of a significant energy gap protecting the insulator. In Fig. 5a we show that for 
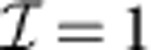
 and 
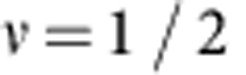
 the gap, computed through exact diagonalization of a small system, is of order 
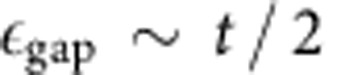
, and is enhanced by the presence of repulsive interaction. Since the gapped phases are crystals with negligible correlation length, the calculation is reliable even if performed for a small system.

The presence of a harmonic confinement, introduced through 
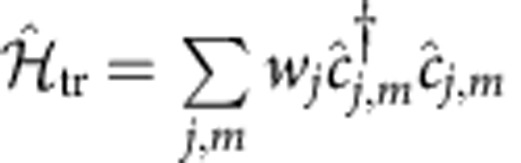
 with 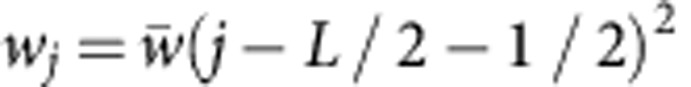 does not hinder the possibility of observing the typical properties of the magnetic crystals discussed so far; in a Thomas–Fermi spirit, they form in definite regions of the trap (see Fig. 5b). In addition, the gap provides a clear temperature window, roughly estimated as 
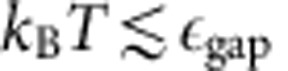
, within which the physics of the state can be observed (*k*_B_ is the Boltzmann constant).

It is natural to envision that the density-spin patterns, which characterize these gapped phases, could be unambiguously revealed through spin-resolved single-site addressing. In addition, several alternative methods based on the coherent interaction between light and the atomic spin have already been proposed to detect many-body phases with spin ordering. We mention, for example, techniques based on Bragg scattering^68^, on polarization spectroscopy^69^ or on spatially resolved imaging via non-resonant light^70^. However, even the less-demanding detection of a gap for such low fillings would constitute a strong hint that the system has been driven into one insulating phase of the hierarchy (1). Concerning the measurement of the helical liquids, the detection of the currents 
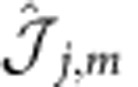
 has been reported in ref. 41 for a bosonic ladder and the same technique can be employed also in our setup.

### Numerical simulations

DMRG is an algorithm that performs a search of the ground state of a Hamiltonian in the space of matrix-product states, a class of states with finite correlations characterized by the so-called bond link, *D* (ref. 50). In the limit *D*=1 matrix-product states are product states, whereas for larger values of *D* more quantum correlations can be described.

We consider chains with open boundaries and length comprised between *L*=96 and 192; setting *D*=200 we are able to describe the correlations in the states with sufficient accuracy. With these parameters the effect of boundaries is irrelevant and the errors on the observables are negligible on the scale of the symbols used in the figures. In the simulations of the gapped crystals, convergence is helped by the quantum numbers related to the conservation of each magnetization ∑_*j*_*n*_*j*,*λ*_; moreover, we find that it can be important to alternate the infinite-size version of the algorithm with the finite-size one.

## Additional information

**How to cite this article:** Barbarino, S. *et al.* Magnetic crystals and helical liquids in alkaline-earth fermionic gases. *Nat. Commun.* 6:8134 doi: 10.1038/ncomms9134 (2015).

## Supplementary Material

Supplementary Information Supplementary Figures 1-6, Supplementary Notes 1-2 and Supplementary References
