# Supplementary material for: Magnetic crystals and helical liquids in alkaline-earth fermionic gases
Source: Nat Commun. 2015 Sep 9;6:8134. doi: 10.1038/ncomms9134 (PMC4579522; doi:10.1038/ncomms9134)
Supplement: Supplementary Information — Supplementary Figures 1-6, Supplementary Notes 1-2 and Supplementary References [file ncomms9134-s1.pdf]

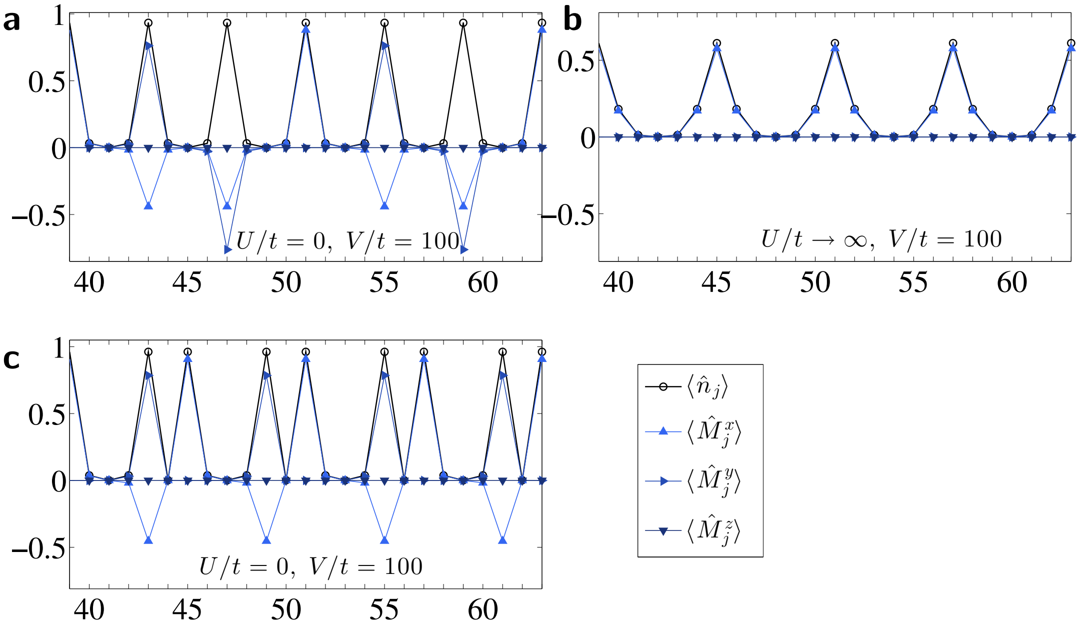

SUPPLEMENTARY FIG. 1. Charge $\langle \hat{n}_i \rangle$ and magnetic order $\langle \hat{M}_i^\alpha \rangle$ of the fractional phases at $\nu = \frac{1}{2}$ (**a**), $\frac{1}{3}$ (**b**) and $\frac{2}{3}$ (**c**) for $k_{\rm SO} = \pi/6$ as obtained from DMRG simulations of a system of length $L = 96$ with $\Omega^{(1)}/t = 1$. For the interaction parameters see the panels. Since the system is a crystal with small boundary effects, we only plot the central part of the system for a better readability.

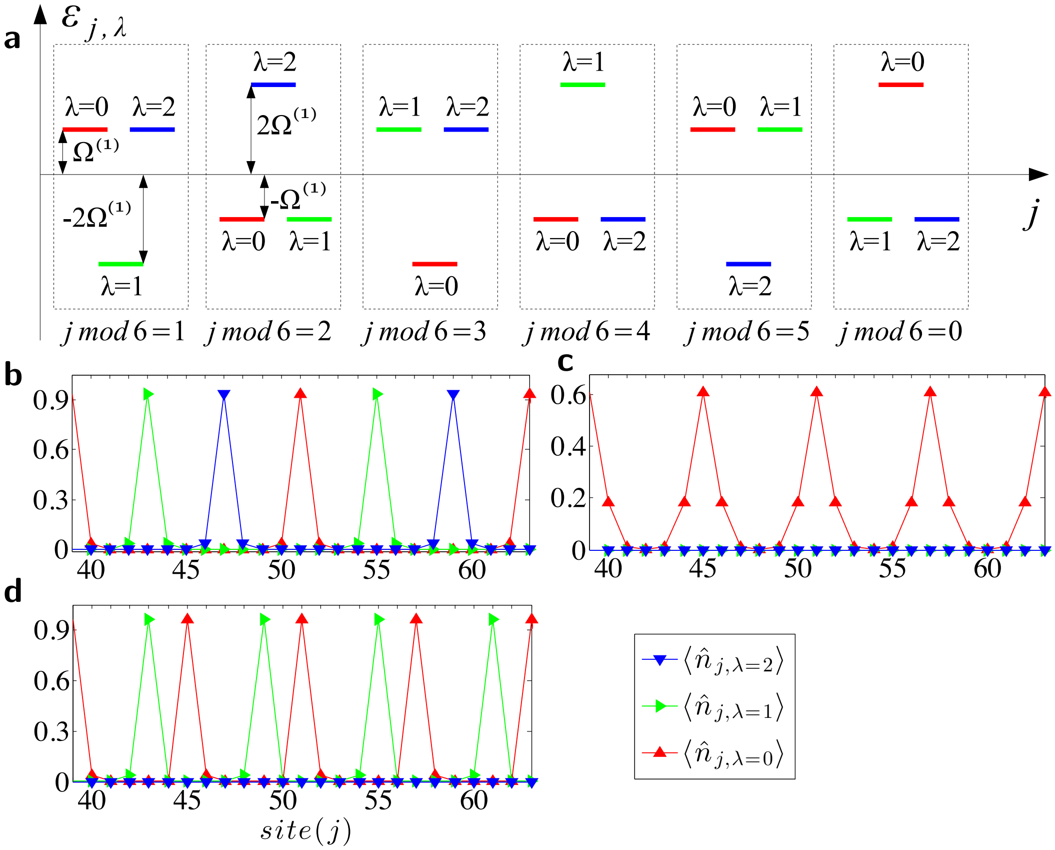

SUPPLEMENTARY FIG. 2. Energy-spin structure $\varepsilon_{j,\lambda}$ for $k_{\rm SO} = \pi/6$ (**a**). Density plots $\langle \hat{n}_{j,\lambda} \rangle$ for fillings $\nu = \frac{1}{2}$ (**b**), $\frac{1}{3}$ (**c**) and $\frac{2}{3}$ (**d**) obtained through DMRG simulations (see the caption of Supplementary Fig. 1 for the parameters).

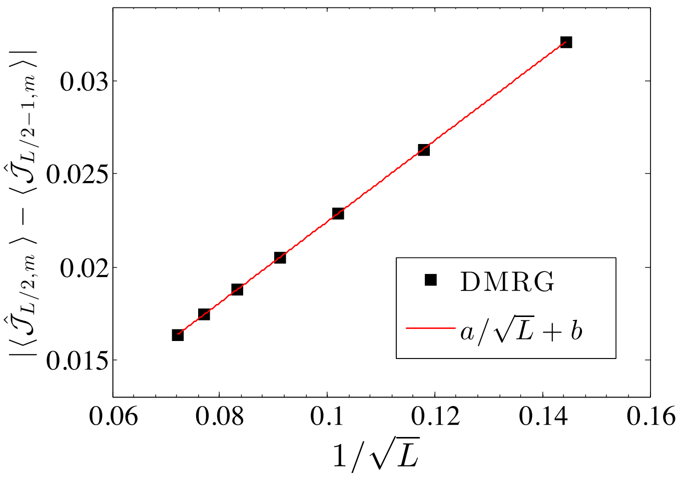

SUPPLEMENTARY FIG. 3. The oscillations of the currents $\langle\hat{\mathcal{J}}_{j,m}\rangle$ vanish in the thermodynamic limit; $a = (0.219 \pm 0.001)$ and $b = (5 \cdot 10^{-5} \pm 4 \cdot 10^{-5})$.

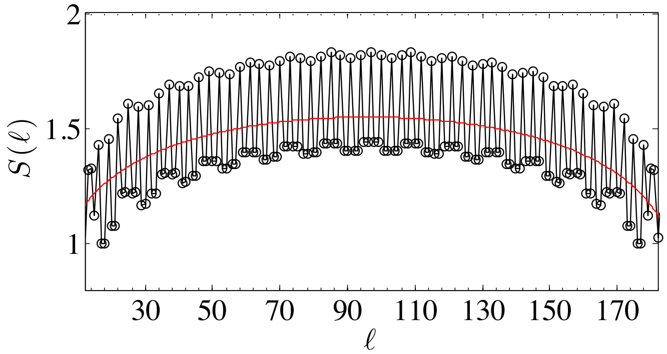

SUPPLEMENTARY FIG. 4. DMRG simulation of the entanglement entropy of the state for $\mathcal{I} = 1$, $L = 192$, $\nu = \frac{1}{4}$, $\Omega^{(1)}/t = 1$, $U/t \to \infty$ for $k_{\mathrm{SO}} = \pi/3$. Thin red line is a fit with the Calabrese-Cardy formula which yields $c = 1.0 \pm 0.1$.

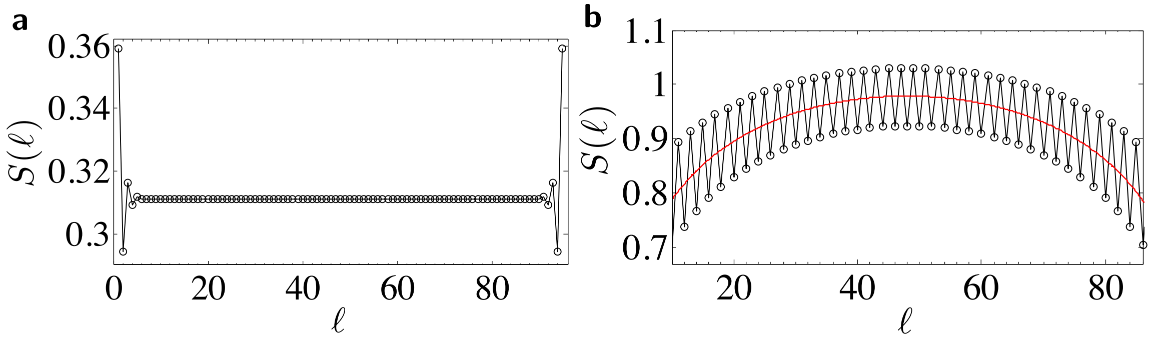

SUPPLEMENTARY FIG. 5. Entanglement entropy $S(\ell)$ of the ground state of a SU(2) fermionic gas for $k_{\mathrm{SO}} = \pi/2$ (**a**) and $k_{SO} = \pi/3$ (**b**); simulation parameters are $\nu = 1/2$, $\Omega^{(1/2)}/t = 1$ and $U/t \to \infty$. The fitted value of the central charge in panel (**b**) is $c = 1.05 \pm 0.15$.

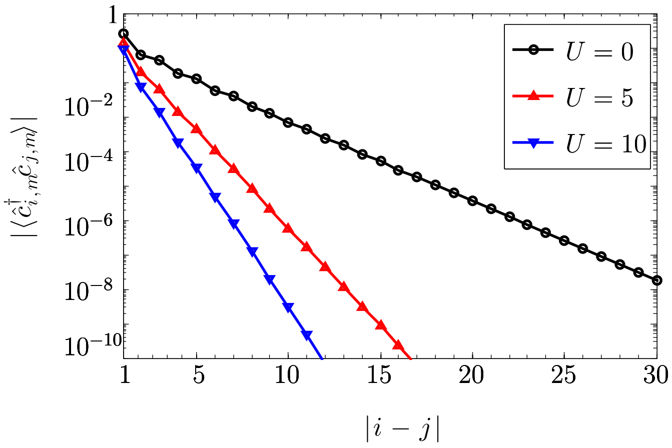

SUPPLEMENTARY FIG. 6. Single-particle correlator $|\langle \hat{c}^{\dagger}_{i,m}\hat{c}_{j,m}\rangle|$ for different values of the interaction strength U; $\nu = 1$, $\Omega^{(1/2)}/t = 1$.

Supplementary Note 1: Additional results for the case $\mathcal{I} = 1$

Numerical results for $k_{\rm SO} = \pi/6$ ***and*** $\hat{\mathcal{H}}_2 \neq 0$***.*** In addition to the data for the fully-gapped phases for $k_{\rm SO} = \pi/3$ discussed in the main text, in Supplementary Fig. 1 we present the density $\langle \hat{n}_j \rangle$ and magnetization profiles $\langle \hat{M}_j^\alpha \rangle$ of the gapped phases for $k_{\rm SO} = \pi/6$ and fillings $\nu = \frac{1}{2}, \frac{1}{3}, \frac{2}{3}$. In Supplementary Fig. 2(**a**) we also show the landscape of the on-site eigenenergies $\varepsilon_{j,\lambda} = 2\Omega^{(\mathcal{I})} \cos\left[\frac{2\pi\lambda}{2\mathcal{I}+1} + 2k_{\rm SO}j\right]$ (note that they explicitly depend on $k_{\rm SO}$, and in the present case they display a six lattice site periodicity), in panels (**b-d**) we show the density profiles in the rotated spin basis, $\langle \hat{n}_{j,\lambda} \rangle$.

Remarkably, they resemble a *diluted* version of the density profiles presented in the main text, suggesting that, at a fixed ν, almost the same physics is obtained by scaling $k_{\rm SO}$ and the number of fermions by the same factor. However, different (e.g. longer range) interactions may be necessary to stabilize phases with the same ν.

Numerical results for $k_{\rm SO} = \pi/3$ ***and*** $\hat{\mathcal{H}}_2 = 0$***.*** We show that the oscillations of the chiral currents vanish in the thermodynamic limit, and can therefore be interpreted as a boundary/finite-size effect (see Supplementary Fig. 3). Conversely, the bulk value of such currents is independent from the system size.

Gapless phases with $\hat{\mathcal{H}}_2 \neq 0$***.*** Finally, we show that the range of the interactions is an essential ingredient to stabilise fully-gapped phases when $\hat{\mathcal{H}}_2 \neq 0$. Supplementary Fig. 4 demonstrates that a simple contact interaction cannot stabilise a gapped phase at $\nu = 1/4$ with $k_{\rm SO} = \pi/3$: indeed, the ground-state von Neumann entanglement entropy displays a dependence on the subsystem size ℓ which is typical of a gapless phase.

Supplementary Note 2: Additional results for the case $\mathcal{I} = 1/2$

Numerical results for $k_{\rm SO} \neq \pi/2$***.*** Let us numerically show that $k_{\rm SO} = \pi/2$ is a necessary condition to obtain crystalline phases for $\mathcal{I} = 1/2$. We consider as an example $\nu = 1/2$ and the interaction $\hat{\mathcal{H}} = U \sum_j \hat{n}_{j,m=1/2}\hat{n}_{j,m=-1/2}$ in the limit $U/t \to \infty$, that stabilises a gapped phase for $k_{SO} = \pi/2$. Consistently, the entanglement entropy of the ground state displays a clear area-law behaviour, see Supplementary Fig. 5(**a**). If $k_{\rm SO}$ is rather tuned to $\pi/3$, under the same conditions the entanglement entropy shows a non-area-law behaviour, which can be fitted with the Calabrese-Cardy formula, signaling the existence of a critical (gapless) ground state, see Supplementary Fig. 5(**b**).

Gapping mechanism. We now discuss the nature of the gapping mechanism responsible for the magnetic crystals studied in the text. The combined action of the Raman coupling $\hat{\mathcal{H}}_1$ with $\hat{\mathcal{H}}_{\rm int}$ gives rise to two commuting Sine-Gordon terms:

$$\int dx \ \cos[\sqrt{2}(q\hat{\phi}_{\rm c} + \hat{\theta}_{\rm s})] + \int dx \ \cos[\sqrt{2}(q\hat{\phi}_{\rm c} - \hat{\theta}_{\rm s})]. \tag{1}$$

Additionally, it is known that when $\nu = 1, \frac{1}{2}, \frac{1}{3}, ...$ the bare interaction $\hat{\mathcal{H}}_{\rm int}$ leads to the additional Mott terms

$$\hat{\mathcal{H}}_{{\rm Mott},\nu} \propto \cos\left(\sqrt{8}\nu^{-1}\hat{\phi}_{\rm c}\right), \tag{2}$$

which constitute a gapping mechanism for the charge degrees of freedom when $K_c < \nu^2$. We now discuss the interplay of the terms (1) and (2) for different fillings ν.

$\nu = 1$. When $U = 0$ and $\Omega \neq 0$ the ground state is fully gapped (band insulator) due to the presence of the terms (1) with $q = 1$ (which are relevant for $K_c < 3$). If $U > 0$, the additional Mott term (2) with $\nu = 1$ (which is relevant for $K_c < 1$) has to be taken into account. As the arguments of these three terms commute, (2) cannot modify the nature of the gapped phase or induce a phase transition (note also that the terms (1) are more relevant). Furthermore, for $U > 0$ we have numerically checked that the gap induced by the terms (1) is enhanced with respect to the non-interacting regime [1], as we can see by studying the correlation length ξ associated to $|\langle \hat{c}^\dagger_{i,m}\hat{c}_{j,m}\rangle| \sim e^{-|i-j|/\xi}$ (see Supplementary Fig. 6) for different values of U. The gapped phase is stabilised by U since the correlation length ξ decreases (and thus the gap is enhanced) when U is increased. The additional fact that for $\Omega = 0$ and $U \gg 0$ the ground state is gapless (only charge degrees of freedom are gapped out) asserts that we are not observing a standard Mott insulator [2, 3].

$\nu = 1/3$. When $U = 0$ and $\Omega \neq 0$ the ground state is gapless. An incompressible phase is stabilised if $\Omega \neq 0$ and $V > 0$ regardless the value of U. In this case we have: the terms (1) with $q = 3$ (relevant if $K_c < 1/3$) and

the Mott term (2) with $\nu = 1/3$ (relevant if $K_c < 1/9$). Taking into account that $K_c > 1/8$, since we only have a nearest-neighbour interaction [4], we obtain that the Mott term is not relevant and it cannot constitute a gapping mechanism. We thus conclude that the incompressible phase we observe is due to the interplay of interactions and magnetic field only. This is further supported by the observation that if we set $\Omega = 0$ we can check numerically that the ground state is gapless and the entanglement entropy scales logarithmically with a central charge $c = 2$ (implying four gapless modes) for all the possible values of U and V.

Supplementary References

[1] Braunecker, B., Japaridze, G. I., Klinovaja, J. & Loss, D. Spin-selective Peierls transition in interacting one-dimensional conductors with spin-orbit interaction. *Phys. Rev. B* **82**, 045127 (2010).

[2] Assaraf, R., Azaria, P., Caffarel, M. & Lecheminant P. Metal-insulator transition in the one-dimensional SU(N) Hubbard model. *Phys. Rev. B* **60**, 2299 (1999).

[3] Manmana, S. R., Hazzard, K. R. A., Chen, G., Feiguin, A. E. & Rey, A. M. SU(N) magnetism in chains of ultracold alkaline-earth-metal atoms: Mott transitions and quantum correlations. *Phys. Rev. A* **84**, 043601 (2011)

[4] Schulz, H. J. Correlation exponents and the metal-insulator transition in the one-dimensional Hubbard model. *Phys. Rev. Lett.* **64**, 2831 (1990).
